# 1-Methyl-1,2,3,4-tetra­hydro­carbolin-2-ium-3-carboxyl­ate

**DOI:** 10.1107/S1600536810002163

**Published:** 2010-01-27

**Authors:** Cheng-Tao Lu, Lin-lin Jing, Hai-Bo Wang, Hai-Feng Tang, Xiao-Li Sun

**Affiliations:** a Department of Chemistry, School of Pharmacy, Fourth Military Medical University, Changle West Road 17, 710032 Xi-An, People’s Republic of China; b Department of Pharmacy, Xijing Hospital, School of Pharmacy, Fourth Military Medical University, Changle West Road 17, 710032 Xi-An, People’s Republic of China

## Abstract

The title compound, C_13_H_14_N_2_O_2_, is a natural product isolated from *Cicer arietinum L*. (chickpea). The benzene ring and pyrrole rings display planar conformations and the piperidine ring has a half-chair conformation. Inter­molecular C—H⋯π inter­actions between a methyl H atom and the pyrrole ring of an adjacent mol­ecule are present in the crystal structure.

## Related literature

For the isolation of the title compound as a natural product, see: Kicha *et al.* (2003 ▶). For the bioactivity of the title compound, see: Adachi *et al.* (1991 ▶); Ogawa & Adachi (1993 ▶).
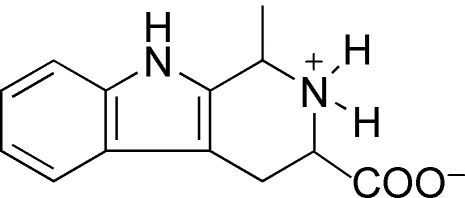


## Experimental

### 

#### Crystal data

C_13_H_14_N_2_O_2_
*M* *_r_* = 230.26
Orthorhombic, 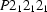

*a* = 4.9772 (8) Å
*b* = 14.520 (2) Å
*c* = 15.307 (2) Å
*V* = 1106.3 (3) Å^3^
*Z* = 4
Mo *K*α radiation
μ = 0.10 mm^−1^
*T* = 296 K
0.32 × 0.23 × 0.13 mm

#### Data collection

Bruker APEXII CCD area-detector diffractometer
Absorption correction: multi-scan (*SADABS*; Bruker, 2000 ▶) *T*_min_ = 0.971, *T*_max_ = 0.988
5528 measured reflections
1166 independent reflections
880 reflections with *I* > 2σ(*I*)
*R*_int_ = 0.053

#### Refinement

*R*[*F* ^2^ > 2σ(*F* ^2^)] = 0.042
*wR*(*F* ^2^) = 0.117
*S* = 1.00
1166 reflections
156 parameters
H-atom parameters constrained
Δρ_max_ = 0.22 e Å^−3^
Δρ_min_ = −0.17 e Å^−3^

### 

Data collection: *APEX2* (Bruker, 2000 ▶); cell refinement: *SAINT* (Bruker, 2000 ▶); data reduction: *SAINT*; program(s) used to solve structure: *SHELXS97* (Sheldrick, 2008 ▶); program(s) used to refine structure: *SHELXL97* (Sheldrick, 2008 ▶); molecular graphics: *ORTEPIII* (Burnett & Johnson, 1996 ▶) and *ORTEP-3 for Windows* (Farrugia, 1997 ▶); software used to prepare material for publication: *SHELXTL* (Sheldrick, 2008 ▶) and *PLATON* (Spek, 2009 ▶).

## Supplementary Material

Crystal structure: contains datablocks I, global. DOI: 10.1107/S1600536810002163/pb2017sup1.cif

Structure factors: contains datablocks I. DOI: 10.1107/S1600536810002163/pb2017Isup2.hkl

Additional supplementary materials:  crystallographic information; 3D view; checkCIF report

## Figures and Tables

**Table 1:** Hydrogen-bond geometry (Å, °) *Cg*1 is the centroid of C1/C6/C7/C11/N1 pyrrole ring.

*D*—H⋯*A*	*D*—H	H⋯*A*	*D*⋯*A*	*D*—H⋯*A*
C10—H10⋯*Cg*1^i^	0.98	2.69	3.644 (3)	165

Symmetry code: (i) 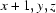.

**Fig. 1.**
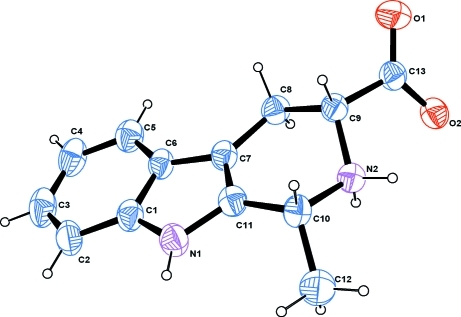
Molecular structure of the title compound (I), showing the atom labeling scheme. Displacement ellipsoids are drawn at the 50% probability level. H atoms are represented as small spheres of arbitrary radii.

## supplementary crystallographic information

### Comment

The title compound is isolated from *Cicer arietinum L*. (Chickpea), It is a precursor of mutagenic *N*-nitroso compounds, which show direct-acting mutagenicity on Salmonlla typhimurium TA 100 (Adachi *et al.*, 1991) and may cause eosinophilia-myalgia syndrome (EMS) associated with ingestion of *L*-tryptophan (Ogawa, 1993).

The benzene ring and pyrrole ring display a planar conformation, and the piperidine ring has a half chair conformation. The molecular packing is stabilized by an intermolecular C–H···π interactions between methyl H atom of piperidine ring pyrrole ring of an adjacent molecule, with a C10–H10···*Cg*1 separation of 2.69 Å (Table 1, *Cg*1 is the centroid of C1—C6—C7—C11—N1 pyrrole ring).

### Experimental

6 kg of the dried chickpea seeds were crushed and refluxed with 70% ethanol. After condensation in a rotary evaporator, 0.6 *L* of a syrupy residue was obtained. The residue was suspended in water and extracted by successive partitioning with petroleum ether, EtOAc, and n-butanol saturated with H_2_O, respectively. The n-butanol portion (60 g) was fractionated over silica gel column eluted with a continuous gradient of CHCl_3_/MeOH/H_2_O (15:1:0–6:4:0.8) mixtures of increasing polarity. Fraction eluted with CHCl_3_/MeOH/H_2_O (7:3:0.5) was combined and afforded crude title compound (1.13 g). Suitable crystal for determining the X-ray stucture was obtained with a further purification by re-crystallization in MeOH/H_2_O (1:1). [α] _D_= 86° (MeOH, 1/4). Spectroscopic analysis: ^1^H NMR(CD_3_OD, 500 MHz): *δ*H 4.71 (1*H*, q, *J*=7 Hz), 3.96 (1*H*, dd, *J*=12, 5.0 Hz), 3.03 (1*H*, m), 3.45 (1*H*, m), 7.47 (1*H*, d, *J*=8.0 Hz), 7.05 (1*H*, m), 7.13 (1*H*, m), 7.34 (1*H*, d, *J*=8.0 Hz), 1.75 (3*H*, d, *J*=7.0 Hz); ^13^C NMR (CD_3_OD, 125 MHz): *δ*c 51.2 (C-10), 59.8 (C-9), 24.3 (C-8), 107.8 (C-7), 127.5 (C-6), 119.2 (C-5), 120.6 (C-4), 123.3 (C-3), 112.3 (C-2), 138.6 (C-1), 131.3 (C-11), 17.1 (C-12), 173.6(C-13).

### Refinement

All H atoms were positioned geometrically and treated as riding with aromatic C—H = 0.93 Å, methine C—H = 0.98 Å, methylene C—H = 0.97Å & methyl C—H = 0.96 Å. The H atom isotropic displacement parameters were fixed; *U*_iso_(aromatic H, methine H) = 1.2 times *U*_eq_ of the parent atom; *U*_iso_(methylene H, methyl H) = 1.5 times *U*_eq_ of the parent atom. Friedel pairs were merged.

### Crystal data

**Table:**

C_13_H_14_N_2_O_2_	*F*(000) = 488
*M**_r_* = 230.26	*D*_x_ = 1.383 Mg m^−^^3^
Orthorhombic, *P*2_1_2_1_2_1_	Mo *K*α radiation, λ = 0.71073 Å
Hall symbol: P 2ac 2ab	Cell parameters from 666 reflections
*a* = 4.9772 (8) Å	θ = 2.7–18.0°
*b* = 14.520 (2) Å	µ = 0.10 mm^−^^1^
*c* = 15.307 (2) Å	*T* = 296 K
*V* = 1106.3 (3) Å^3^	Needle, colorless
*Z* = 4	0.32 × 0.23 × 0.13 mm

### Data collection

**Table:**

Bruker APEXII CCD area-detector diffractometer	1166 independent reflections
Radiation source: fine-focus sealed tube	880 reflections with *I* > 2σ(*I*)
graphite	*R*_int_ = 0.053
phi and ω scans	θ_max_ = 25.1°, θ_min_ = 1.9°
Absorption correction: multi-scan (*SADABS*; Bruker, 2000)	*h* = −5→5
*T*_min_ = 0.971, *T*_max_ = 0.988	*k* = −17→15
5528 measured reflections	*l* = −16→18

### Refinement

**Table:**

Refinement on *F*^2^	Secondary atom site location: difference Fourier map
Least-squares matrix: full	Hydrogen site location: inferred from neighbouring sites
*R*[*F*^2^ > 2σ(*F*^2^)] = 0.042	H-atom parameters constrained
*wR*(*F*^2^) = 0.117	*w* = 1/[σ^2^(*F*_o_^2^) + (0.074*P*)^2^] where *P* = (*F*_o_^2^ + 2*F*_c_^2^)/3
*S* = 1.00	(Δ/σ)_max_ < 0.001
1166 reflections	Δρ_max_ = 0.22 e Å^−^^3^
156 parameters	Δρ_min_ = −0.17 e Å^−^^3^
0 restraints	Extinction correction: *SHELXL97* (Sheldrick, 2008), Fc^*^=kFc[1+0.001xFc^2^λ^3^/sin(2θ)]^-1/4^
Primary atom site location: structure-invariant direct methods	Extinction coefficient: 0.026 (6)

### Special details

**Table:**

Geometry. All e.s.d.'s (except the e.s.d. in the dihedral angle between two l.s. planes) are estimated using the full covariance matrix. The cell e.s.d.'s are taken into account individually in the estimation of e.s.d.'s in distances, angles and torsion angles; correlations between e.s.d.'s in cell parameters are only used when they are defined by crystal symmetry. An approximate (isotropic) treatment of cell e.s.d.'s is used for estimating e.s.d.'s involving l.s. planes.
Refinement. Refinement of *F*^2^ against ALL reflections. The weighted *R*-factor *wR* and goodness of fit *S* are based on *F*^2^, conventional *R*-factors *R* are based on *F*, with *F* set to zero for negative *F*^2^. The threshold expression of *F*^2^ > σ(*F*^2^) is used only for calculating *R*-factors(gt) *etc*. and is not relevant to the choice of reflections for refinement. *R*-factors based on *F*^2^ are statistically about twice as large as those based on *F*, and *R*- factors based on ALL data will be even larger.

### Fractional atomic coordinates and isotropic or equivalent isotropic displacement parameters (Å^2^)

**Table:**

	*x*	*y*	*z*	*U*_iso_*/*U*_eq_	
N1	0.3184 (7)	0.56276 (18)	0.71317 (15)	0.0402 (7)	
H1	0.2878	0.5045	0.7104	0.048*	
N2	−0.0123 (6)	0.67564 (14)	0.90152 (14)	0.0319 (6)	
H2A	−0.1486	0.6668	0.9393	0.038*	
H2B	0.1421	0.6729	0.9320	0.038*	
O2	−0.0135 (5)	0.82385 (13)	1.00915 (13)	0.0390 (6)	
O1	−0.2002 (7)	0.91384 (15)	0.90704 (15)	0.0581 (8)	
C1	0.4997 (7)	0.6110 (2)	0.66226 (19)	0.0382 (8)	
C2	0.6786 (8)	0.5800 (3)	0.59940 (19)	0.0476 (9)	
H2	0.6826	0.5183	0.5830	0.057*	
C3	0.8495 (8)	0.6429 (3)	0.5621 (2)	0.0539 (11)	
H3	0.9745	0.6232	0.5209	0.065*	
C4	0.8388 (8)	0.7364 (3)	0.5849 (2)	0.0503 (9)	
H4	0.9523	0.7782	0.5574	0.060*	
C5	0.6633 (8)	0.7669 (3)	0.6473 (2)	0.0441 (9)	
H5	0.6573	0.8290	0.6622	0.053*	
C6	0.4939 (7)	0.7043 (2)	0.68836 (19)	0.0348 (7)	
C7	0.2982 (7)	0.7105 (2)	0.75634 (17)	0.0343 (8)	
C8	0.2132 (7)	0.7924 (2)	0.8087 (2)	0.0369 (8)	
H8A	0.1759	0.8437	0.7699	0.044*	
H8B	0.3574	0.8105	0.8477	0.044*	
C9	−0.0383 (7)	0.7696 (2)	0.86201 (19)	0.0325 (8)	
H9	−0.1924	0.7690	0.8222	0.039*	
C10	−0.0145 (7)	0.59951 (19)	0.83517 (18)	0.0345 (8)	
H10	−0.1902	0.5989	0.8062	0.041*	
C11	0.1945 (8)	0.6245 (2)	0.76945 (19)	0.0348 (8)	
C12	0.0263 (10)	0.5078 (2)	0.8807 (2)	0.0550 (11)	
H12A	0.1971	0.5078	0.9098	0.082*	
H12B	0.0219	0.4591	0.8383	0.082*	
H12C	−0.1142	0.4986	0.9228	0.082*	
C13	−0.0897 (7)	0.8423 (2)	0.9318 (2)	0.0355 (8)	

### Atomic displacement parameters (Å^2^)

**Table:**

	*U*^11^	*U*^22^	*U*^33^	*U*^12^	*U*^13^	*U*^23^
N1	0.0454 (19)	0.0379 (14)	0.0374 (15)	−0.0017 (15)	0.0046 (15)	−0.0056 (11)
N2	0.0258 (15)	0.0372 (13)	0.0326 (13)	−0.0020 (13)	−0.0009 (14)	−0.0002 (10)
O2	0.0340 (14)	0.0448 (13)	0.0381 (12)	0.0015 (11)	0.0012 (12)	−0.0039 (10)
O1	0.076 (2)	0.0457 (14)	0.0523 (14)	0.0215 (15)	0.0009 (15)	0.0018 (12)
C1	0.035 (2)	0.0501 (19)	0.0292 (16)	0.0034 (19)	−0.0021 (17)	−0.0026 (13)
C2	0.048 (2)	0.061 (2)	0.0336 (17)	0.011 (2)	0.0031 (19)	−0.0081 (17)
C3	0.038 (2)	0.087 (3)	0.037 (2)	0.005 (2)	0.0069 (19)	−0.0038 (19)
C4	0.034 (2)	0.079 (3)	0.0377 (18)	−0.009 (2)	0.0057 (19)	0.0038 (18)
C5	0.038 (2)	0.056 (2)	0.0382 (17)	−0.0014 (19)	0.0014 (18)	0.0012 (15)
C6	0.0291 (18)	0.0470 (18)	0.0283 (14)	0.0000 (17)	−0.0003 (16)	−0.0008 (13)
C7	0.032 (2)	0.0406 (17)	0.0303 (16)	−0.0006 (15)	−0.0002 (16)	−0.0013 (12)
C8	0.033 (2)	0.0390 (17)	0.0383 (16)	−0.0018 (15)	0.0021 (16)	0.0035 (13)
C9	0.026 (2)	0.0388 (17)	0.0324 (15)	0.0009 (14)	0.0005 (15)	0.0017 (13)
C10	0.0354 (19)	0.0374 (17)	0.0306 (16)	−0.0050 (17)	0.0013 (17)	−0.0018 (12)
C11	0.0312 (19)	0.0412 (18)	0.0319 (15)	0.0003 (15)	−0.0038 (15)	−0.0034 (13)
C12	0.073 (3)	0.041 (2)	0.051 (2)	−0.001 (2)	0.009 (2)	0.0002 (15)
C13	0.0307 (19)	0.0395 (18)	0.0363 (18)	−0.0005 (15)	0.0043 (15)	0.0011 (14)

### Geometric parameters (Å, °)

**Table:**

N1—C1	1.383 (4)	C5—C6	1.390 (5)
N1—C11	1.388 (4)	C5—H5	0.9300
N1—H1	0.8600	C6—C7	1.428 (5)
N2—C9	1.498 (4)	C7—C11	1.367 (4)
N2—C10	1.501 (3)	C7—C8	1.495 (4)
N2—H2A	0.9000	C8—C9	1.530 (5)
N2—H2B	0.9000	C8—H8A	0.9700
O2—C13	1.272 (4)	C8—H8B	0.9700
O1—C13	1.234 (4)	C9—C13	1.523 (4)
C1—C2	1.386 (5)	C9—H9	0.9800
C1—C6	1.412 (4)	C10—C11	1.492 (4)
C2—C3	1.373 (5)	C10—C12	1.516 (4)
C2—H2	0.9300	C10—H10	0.9800
C3—C4	1.404 (5)	C12—H12A	0.9600
C3—H3	0.9300	C12—H12B	0.9600
C4—C5	1.368 (5)	C12—H12C	0.9600
C4—H4	0.9300		
			
C1—N1—C11	108.2 (3)	C7—C8—C9	110.2 (3)
C1—N1—H1	125.9	C7—C8—H8A	109.6
C11—N1—H1	125.9	C9—C8—H8A	109.6
C9—N2—C10	113.4 (2)	C7—C8—H8B	109.6
C9—N2—H2A	108.9	C9—C8—H8B	109.6
C10—N2—H2A	108.9	H8A—C8—H8B	108.1
C9—N2—H2B	108.9	N2—C9—C13	111.3 (2)
C10—N2—H2B	108.9	N2—C9—C8	110.0 (3)
H2A—N2—H2B	107.7	C13—C9—C8	111.2 (3)
N1—C1—C2	130.2 (3)	N2—C9—H9	108.1
N1—C1—C6	108.2 (3)	C13—C9—H9	108.1
C2—C1—C6	121.4 (3)	C8—C9—H9	108.1
C3—C2—C1	118.1 (3)	C11—C10—N2	105.8 (2)
C3—C2—H2	121.0	C11—C10—C12	115.5 (3)
C1—C2—H2	121.0	N2—C10—C12	109.5 (2)
C2—C3—C4	121.1 (3)	C11—C10—H10	108.6
C2—C3—H3	119.5	N2—C10—H10	108.6
C4—C3—H3	119.5	C12—C10—H10	108.6
C5—C4—C3	120.8 (4)	C7—C11—N1	109.4 (3)
C5—C4—H4	119.6	C7—C11—C10	125.8 (3)
C3—C4—H4	119.6	N1—C11—C10	124.9 (3)
C4—C5—C6	119.4 (3)	C10—C12—H12A	109.5
C4—C5—H5	120.3	C10—C12—H12B	109.5
C6—C5—H5	120.3	H12A—C12—H12B	109.5
C5—C6—C1	119.1 (3)	C10—C12—H12C	109.5
C5—C6—C7	134.5 (3)	H12A—C12—H12C	109.5
C1—C6—C7	106.3 (3)	H12B—C12—H12C	109.5
C11—C7—C6	107.8 (3)	O1—C13—O2	126.5 (3)
C11—C7—C8	122.8 (3)	O1—C13—C9	116.3 (3)
C6—C7—C8	129.4 (3)	O2—C13—C9	117.2 (3)
			
C11—N1—C1—C2	177.1 (3)	C10—N2—C9—C8	−67.4 (3)
C11—N1—C1—C6	1.3 (4)	C7—C8—C9—N2	42.5 (3)
N1—C1—C2—C3	−176.0 (3)	C7—C8—C9—C13	166.2 (3)
C6—C1—C2—C3	−0.7 (5)	C9—N2—C10—C11	52.2 (3)
C1—C2—C3—C4	−1.8 (5)	C9—N2—C10—C12	177.2 (3)
C2—C3—C4—C5	2.2 (6)	C6—C7—C11—N1	0.7 (4)
C3—C4—C5—C6	0.0 (6)	C8—C7—C11—N1	−178.1 (3)
C4—C5—C6—C1	−2.4 (5)	C6—C7—C11—C10	179.4 (3)
C4—C5—C6—C7	177.5 (4)	C8—C7—C11—C10	0.6 (5)
N1—C1—C6—C5	179.0 (3)	C1—N1—C11—C7	−1.3 (4)
C2—C1—C6—C5	2.8 (5)	C1—N1—C11—C10	−180.0 (3)
N1—C1—C6—C7	−0.9 (3)	N2—C10—C11—C7	−19.6 (4)
C2—C1—C6—C7	−177.1 (3)	C12—C10—C11—C7	−140.9 (4)
C5—C6—C7—C11	−179.8 (4)	N2—C10—C11—N1	158.9 (3)
C1—C6—C7—C11	0.1 (4)	C12—C10—C11—N1	37.6 (4)
C5—C6—C7—C8	−1.1 (6)	N2—C9—C13—O1	−156.8 (3)
C1—C6—C7—C8	178.8 (3)	C8—C9—C13—O1	80.3 (4)
C11—C7—C8—C9	−12.0 (4)	N2—C9—C13—O2	23.7 (4)
C6—C7—C8—C9	169.5 (3)	C8—C9—C13—O2	−99.2 (3)
C10—N2—C9—C13	168.9 (3)		

### Hydrogen-bond geometry (Å, °)

**Table:**

*Cg*1 is the centroid of C1/C6/C7/C11/N1 pyrrole ring.

**Table:**

*D*—H···*A*	*D*—H	H···*A*	*D*···*A*	*D*—H···*A*
C10—H10···Cg1^i^	0.98	2.69	3.644 (3)	165

Symmetry codes: (i) *x*+1, *y*, *z*.
